# Claiming control: cooperation with return as a condition for social benefits in Austria and the Netherlands

**DOI:** 10.1186/s40878-018-0085-3

**Published:** 2018-09-04

**Authors:** Sieglinde Rosenberger, Sabine Koppes

**Affiliations:** 1 University of Vienna, Vienna, Austria; 2 Research Fellow University of Vienna, Vienna, Austria

**Keywords:** Cooperation with return, Migrants in limbo, Social benefits, Migration-social policy nexus, Austria, The Netherlands

## Abstract

Theoretically embedded in the migration/social policy nexus, this paper investigates cooperation with return (CWR) as a policy tool to remove practical deportation barriers for third-country nationals pending removal. Based on legal and policy documents and expert interviews with stakeholders in Austria and the Netherlands, the paper asks how CWR is implemented and what influence it has, both on migration control aims and on access to social rights. We argue that the politicization of the issue and diverging interests between policy networks of welfare and migration affect the regulation and implementation of the tool. By comparing the use of CWR within two country contexts, the analysis presented here adds valuable insights on features of governmental instruments in response to the “deportation gap”. The paper further adds to the literature on sanction-oriented, personalized migration policies.

## Introduction

For some European countries, the year 2015 brought an unprecedented increase in asylum applications and, eventually, the prominence of the “refugee crisis” within public discourse. In response, the themes of migration control and return policies were re-entered in the political and public agenda. Broadly speaking, three policy approaches to cope with accelerated migration gained in relevance: legal and practical restrictions to the entry of asylum seekers, a strengthening of return enforcement, and restrictions in social welfare for certain groups of migrants. In particular, the forced or “voluntary” removal of rejected asylum seekers became a formula for handling the refugee crisis. To this end, policy makers at European and national levels prioritized return efforts and took also action to facilitate the return of so-called non-removals (DeBono, 2016; Lutz, 2017; Weatherhead, 2016).

Forced return has always been a contested part of the politics of migration control. Not only must returning instruments comply with human rights provisions, their implementation also faces technical and practical constraints. As a result of impediments and international safeguards, some migrants without a residency status cannot be removed from the state’s territory. Practical barriers concern the relations between the host country and the country of origin (e.g. lack of readmission agreements) and are deemed to lie partly in the capacity of the individual (e.g. non-cooperation with identification papers and travel documents). However, liberal states have only limited tools to eliminate such practical or technical barriers (Trauner & Kruse, 2008). Against this background, countries have turned to *cooperation with return* (CWR) as a policy tool to regulate better presence and access to social rights. Only once active cooperation with the return has been demonstrated and confirmed by the authorities is the migrant treated as non-removable and earns the right to reside for a certain period of time. In addition, some countries couple this requirement for remaining with access to social services, on the assumptions that this will make return strategies more efficient. In other words: Migration control is carried out with the help of conditional access to basic social rights. The tool combines a duty with a right, *if* a person cooperates with return processes, *then* he/she may be granted stay and welfare support. This approach relates directly to the so-called migration/social policy nexus which serves as the main analytical frame for discussing CWR as a policy tool (Bommes & Geddes, 2000; Goldring & Landolt, 2013; Guiraudon & Joppke, 2001; Wright, 2016).

Analytically, the paper is informed by policy analysis literature which deals with policy instruments or policy tools^1^ as devices that governments use to implement policies (Howlett & Ramesh, 2003). Migration studies dealing with social welfare tools often refer to the stratified-rights approach (Morris, 2010). In this paper, we deal with CWR as a policy instrument of migration control, which is simultaneously employed as a prerequisite for gaining the precarious status of being non-removable, and accessing housing and social allowances. We expect that social benefits, coupled with cooperation in the return procedures, are intended as the “inducements” (Howlett & Ramesh, 2003, p. 90) to attain migration control aims rather than cuts in welfare expenses. Nevertheless, strict implementation of the instrument may lead to problematic, unintended consequences in social welfare such as homelessness and destitution (Spencer, 2016). Within this tension between migration control and welfare aims, we trace how these policy fields work together or against each other (Rhodes, 2008).

The analysis focuses on third-country nationals who are eligible for removal according to the host state, but are not removed for various reasons (Mananashvili, 2017; Wong, 2015). In general, the group of non-removable returnees consists of undocumented migrants as well as third-country nationals who have received a (final) negative decision on their asylum application. In the interest of conceptual clarity, the analysis here sets aside other administrative categories and covers only rejected asylum seekers pending deportation, as they are recorded by the state (Heegaard Bausager, Møller, & Ardittis, 2013) and therefore most likely to be affected by the policy tool. Hence, only the group that is visible to the state is included in the empirical study.

We refer to this group as *migrants in limbo* to express that the individuals are living in a state of extreme legal and social uncertainty (Goldring & Landolt, 2013, p. 12). To date there are only a few studies that have described the legal and social situation of third-country nationals pending deportation in Europe (Bolhuis, Battjes, & Wijk, 2017; Cantor, Wijk, Singer, & Bolhuis, 2017; Fundamental Rights Agency (FRA), 2013; Heegaard Bausager et al., 2013; Rosenberger & Küffner, 2016; Spencer, 2016). According to international and European legal frameworks, the mere presence of these individuals urges the liberal state to take some responsibility for their well-being. More precise, the right to an adequate standard of living is to be upheld regardless of one’s residency status (see Committee on Economic, Social and Cultural Rights (CESCR), 2017; (FRA, 2013)). Though, nation states provide access to social benefits in a highly conditional manner and depending on the fulfilment of several requirements – one of them is cooperation with return procedures.

Goldring and Landolt (2015) have investigated the production of social membership for irregular migrants in Canada. For the Netherlands, scholars have analysed the dynamics and struggles between national policies of welfare exclusion of irregular migrants and the strategies municipalities have developed to cope with exclusionary regulations (Engbersen & Broeders, 2009; Kos, Maussen, & Doomernik, 2015; Leerkes, 2016). Beside these studies, we do not have much knowledge on the implementation of policy tools linking immigration control aims with welfare entitlements (see DeBono, Rönneqvist, & Magnusson, 2015). In particular, there is a large gap in research investigating the use and effects of policy inducements in the case of rejected asylum seekers pending deportation.

Against this research background, we raise the following questions: Which role does CWR play for both the politics of deportation and for selective access to social rights? Which political tensions go along with the use of cooperation as a condition for social benefits? Also, how can we understand cross-country differences in the use of CWR? To answer these questions, the paper uses two countries, Austria and the Netherlands, as case studies for an in-depth investigation. We carried out interviews with a wide variety of stakeholders in the field and an analysis of legal and policy documents, which yielded insights into the characteristics and relevance of the tool – it is vaguely defined, opens up leeway for street-level bureaucrats and causes conflicts between political levels as well as insecurity and indeterminacy for individuals. We show that in both countries CWR serves symbolic aims rather than being effective in increasing return rates or cutting welfare spending. The more pronounced role of CWR in the Netherlands can be attributed to the different extent of politicization of irregular migration and welfare. Our analysis thus contributes to the knowledge on sanction-oriented governmental instruments targeting individuals in response to the migration control dilemma (Chauvin & Garcés-Mascareñas, 2012; Engbersen & Broeders, 2009).

## Theoretical and analytical approaches

Liberal nation states show a tendency to intensify border policies after entry to control or manage migrant flows. At the same time, they often do not have the power to enforce them and meet the apprehensions of those sections of society who demand a hardening of immigration control. Consequently, discursive, implementation and efficacy gaps may occur (Czaika & de Haas, 2013). Various reasons have been identified why and to which extent a liberal state’s capacity to control international migration is constrained. Frequently mentioned are consequences of universal human rights regimes, which protect and accommodate migrants beyond national citizenship (Bommes & Geddes, 2000; Guiraudon & Lahav, 2006).

Considering these migration control gaps, the question is which policy responses the liberal state can administer to deal with them. A common response to the control dilemma is located in the migration/social policy nexus (Sainsbury, 2006; Wright, 2016). The main objective is to have migration and social policy instruments connected to serve control aims. However, this nexus is built by two policy fields with very different actors, networks, logics and aims. Social policies belong to the category of redistributive policies and return policies are a type of regulative policy (Rhodes, 2008). The overall aim of social policies is to foster the well-being of individuals and social justice within society, while the key aim of migration control is to prevent “all unlawful migration”, as Wong states (2015, p. 83). Both Sainsbury (2006) and Wright (2016) notice a tension inherent to immigration policy on the one side and social policy on the other. They argue that restrictive migration approaches are intended to protect the generosity of the national welfare state. In the face of extensive migration flows, however, it seems more important for governments to secure borders and state sovereignty. In this context, social policy restrictions are designed to de-incentivize immigration and stay. As a result, policy responses in the realm of restrictive and selective access to social welfare also serve a political aim as large parts of the electorate prefer lower levels of social services for non-nationals (Van Oorschot, 2006).

To investigate the characteristics and functioning of CWR as a policy tool, we draw on public policy literature. Policy scholars refer to tools or instruments as means to secure certain aims and concrete outputs of political authorities. Policy instruments are targeted towards individuals or collectives and are used to reward or punish behaviour, to certify or sanction certain types of policy-relevant behaviour (Howlett & Ramesh, 2003; Linder & Peters, n.d.). More specifically, in migration and citizenship studies, policy instruments are often conceptualized as part of the conditionality of presence and access. Dwyer and Brown point out that in most welfare states access to welfare entitlements depends on certain duties and patterns of behaviour that an individual must agree to meet (Dwyer & Brown, 2005, p. 370). Goldring and Landolt (2015) present an analytical framework for the study of the production of membership of irregular migrants and define conditionality as follows:

Conditionality denotes the material and discursive conditions that must be met to acquire and exercise the formal and substantive right to remain present within a national territory and/or to access entitlements and social goods, including the labor market. (Goldring & Landolt, 2015, p. 857)

Numerous studies on migrants’ access to welfare services underline that benefits are made conditionally and are fragmented, dependent on administrative categorization and individual good behaviour (Chauvin & Garcés-Mascareñas, 2012; Morris, 2010). Discretionary power and judgements by street-level bureaucrats and case workers play an essential role in access to services (Molander, 2017). As this highlighted literature demonstrates, discretion and conditionality of access to social welfare in relation to migration control is not new. However, this paper will show that the contradictive aims of the two policy fields concerned in CWR have a particular impact on the practical implementation of the tool.

For tracing the role, characteristics and functioning of the tool, we developed a framework on the basis of policy analysis literature which differentiates three features: 1) The *substance*. What kind and degree of cooperation is demanded legally and in practice? 2) The *actors and processes*. Who checks and decides whether a migrant is sufficiently cooperating? 3) The *effects*. Does cooperation affect return enforcement and/or the scope of social benefits? (on policy dimensions see Howlett & Ramesh, 2003, p. 90). Prior to providing empirical information on these features, we present contextual information on the countries studied, as well as on methods and data.

## Study countries and methods

We chose Austria and the Netherlands for a comparative analysis to get a better understanding of the nature, implementation and effects of the cooperation tool. The two countries have some macro-factors in common and allow us to make an in-depth study of the nature and implementation of CWR. First, our study countries share the same legal European framework regarding return and reception provisions. As European Member States, they are bound to uphold minimum standards under the Reception Directive 2013/33/EC and the Return Directive 2008/115/EC. The latter sets out that all Member States have to grant at least emergency health care and access to basic education. Alongside this regulation, under the 2015 European Agenda on Migration, the European Commission strives to increase the enforcement rate of return decisions and to increase agreements with third countries to facilitate return, signalling a growing support of return policies in European Member States (Lutz, 2017).

Second, in terms of competences and powers in migration control, the national level dominates sub-national authorities in both countries. In Austria, the power to issue and enforce return decisions remains solely with the federal state (Ministry of the Interior, Federal Agency for Immigration and Asylum/BFA). In turn, federal authorities and the authorities of the provinces have executive competences and financial obligations for the welfare of (rejected) asylum seekers. This institutional design of shared responsibilities in welfare creates a situation in which the rules are the same across the country, but they do not all apply in the same way. In practice, some provinces act more restrictively, others more supportively (Rosenberger & König, 2011). A similar structure applies to the Netherlands, where powers in migration and asylum also rest with the national government (State Secretary of Security and Justice). Certain responsibilities pertaining to welfare, such as housing, are delegated to local government, giving the local authorities a degree of leeway in the interpretation of these policies (Kos et al., 2015).

Third, both countries experience what is called the deportation gap (Gibney, 2008, p. 149). This term designates the mismatch between the number of people eligible for removal by the state and the number of people that a state actually removes. EUROSTAT uses a different terminology and speaks about effective returns and defines this term as the ratio between third-country nationals ordered to leave and third-country nationals returned following an order to leave. In 2016, effective returns reached 50% (almost half a million non-EU citizens were ordered to leave the EU, but less than 230,000 are known to have effectively left). In Austria, the return rate is close to the EU average, in the Netherlands the rate is under 40% (Mananashvili, 2017). The total rate in Austria is clearly higher than in the Netherlands, which also reflects higher numbers of asylum applications in recent years in Austria compared to the Netherlands.

The two countries differ in the extent and the way irregular migration and the “deportation gap” are politicized. Rosenberger and Ruedin (2017) show in a comparative study on the politicization of immigrant groups that in Austria it is the group of asylum seekers that has been constantly politicized, but less the category of irregular migrants and their stay and social rights.

The data for this paper were compiled within a comparative research project investigating the political and social conditions of access to social rights for non-removable migrants.^2^ The data collection includes a range of semi-structured qualitative expert interviews with policy makers, government officials at national and sub-national levels, and other stakeholders, such as academics, lawyers and NGO staff. The interviewees are experts on access to social benefits for migrants in limbo from the perspectives of national government, local government and NGOs. They provide insights into the use and effects of the policy tool from diverse institutionary roles. A total of 21 interviews were held in the Netherlands and 25 in Austria between June 2016 to July 2017. The interviews were transcribed verbatim and analysed by the summative content analysis method (Hsieh & Shannon, 2005). This method of analysis using coding software allowed for detailed and structured insights into the knowledge, reasoning and views of different stakeholders. In addition to the interviews, primary and secondary documents, such as legal commentaries, legislative texts, laws and parliamentary documents were included. Further, decisions by international institutions as well as statements by leading human rights institutions were considered.

We now proceed by mapping out empirical insights into CWR, its substance, nature and tendencies separately for each country.

## Cooperation with return: the case of Austria

In Austria, the *obligation to cooperate (Mitwirkungspflicht)* with return authorities is an essential condition for being authorized as (temporary) non-removable, being granted the toleration status and access to certain social benefits. Very recently, the amendments of the Aliens Act (*Fremdenrechtsgesetz*) of June 2017 include a reference to non-cooperation with return, which in case of non-compliance may lead to detention and high penalties. In general terms, this policy is sanction-oriented, it is the precondition for entitlements and nowadays moves on to punishments.

### Cooperation for remaining and toleration status

Cooperation with return is necessary to qualify as “non-removable”. Only if rejected asylum seekers are considered cooperative can they potentially receive a formal toleration status (*Duldungskarte*). According to the law, CWR is the main condition for being granted toleration status. Specifically, migrants applying for toleration status have to demonstrate: 1) that they cooperate with the aliens authorities to provide correct identity information and do not veil his/her identity, 2) that they have approached their embassy to produce valid travel certificates (National Council, 2005).

As said above, cooperation is fully necessary for obtaining the toleration status, but it is far from being a sufficient condition. Every year roughly 200 to 400 toleration cards are actually issued by the federal authorities (Federal Ministry of the Interior, 2016). This low numbers tell us that many migrants pending deportation fail to be granted toleration status. The gatekeepers for toleration status are officials of the Ministry of the Interior, via its sub-ordinated agency, the BFA. Administrative authorities denying the toleration card argue that applicants have not cooperated fully, they have provided false information (Head of legal counseling for asylum seekers at an NGO in Vienna, personal communication, 14 September 2016). Nevertheless, decisions on toleration status depend on subjective assessments. To illustrate, in one of the interviews a high official in the Austrian Migration Agency states his definition of cooperation as follows: “…there are necessary steps to get travel documents, [therefore he/she] either cooperates or doesn’t cooperate” (High level bureaucrat at the national migration agency, personal communication, n.d.). However, he relativizes shortly after that “it can certainly be a discussion in the individual case” (High level bureaucrat at the national migration agency, personal communication n.d.). These statements underline that discretionary power of street-level bureaucrats (see Lipsky, 2010; Molander, 2017) plays a role in the assessments of cooperation with return.

### Cooperation for social benefits

The Basic Welfare Support Agreement *(Grundversorgungsvereinbarung)*, adopted in 2004 between the federal state and the nine provinces, defines certain groups as in need of protection: asylum seekers, asylum holders, displaced persons, as well as “aliens without a residence permit, who for legal or factual reasons cannot be removed” (National Council, 2004). All these groups are legally entitled to a minimum standard of support, including housing, financial allowances, health insurance and basic education. The two main conditions to be met are financial hardship and active cooperation with return procedures. Legally, the obligation to cooperate pertains to financial assistance and accommodation. For services such as primary health care and basic education, cooperation is not a direct and necessary requirement (Provincial Minister for integration, personal communication, 7 June 2017).

According to the Basic Welfare Support Agreement, rejected asylum applicants have to be deemed non-removable “for legal or factual reasons” to be allowed access to basic care. In any case, reasons for postponement must not lie within the person’s own responsibility. When the migrant refuses to cooperate then he/she runs the risk of being excluded, not only from basic care, but also to be detained in detention centres. The individual has to cooperate in clearing all practical barriers towards removal which are rooted in his/her behaviour. According to the law and to interviews with representatives of state authorities and advocacy groups, the individual has to comply with the following rules: The non-removed person has to establish contact with migration control authorities, specifically with the aliens police. Their activities must be actively supported by delivering identification documents (name and country of origin). Cooperation also includes handing out a passport photograph and not to refuse signatures. The law, however, can never be clear-cut in defining the scope of the required cooperation. Discretionary assessments by street-level bureaucrats whether the criterion has been sufficiently demonstrated are necessary and crucial. Moreover, the given embassies play a role, sometimes in favour, sometimes to the detriment of the migrant (Head of legal counseling for asylum seekers at an NGO in Vienna, personal communication, 14 September 2016).

At province level, cooperation has become important only in the transposition of the Return Directive 2013 into provincial law. Seven out of nine provinces have newly inserted the obligation to cooperate with obtaining travel documents into the state laws on basic care support.^3^ Only law makers in the provinces of Vienna and Vorarlberg refrained from considering it a condition for access to welfare support. In practice, rather than actively contributing to the procedure by the aliens police, a non-removed person must make oneself regularly accessible for the authorities (High level bureaucrat at Viennese agency for social policy, personal communication, 3 October 2016; Case worker at an NGO in Vienna, personal communication, 24 October 2016). In the cases cooperation is interpreted as visibility.

Checking cooperation is a difficult administrative procedure with inherent tensions, inconsistencies and discretion. Not surprisingly, it is return officers and not welfare authorities, who control whether CWR is successfully met by the migrant in question. Secondly, control of cooperation is not delegated to sub-national parties, but it remains with the federal authorities. Practically, the administrative procedure goes like this: Officers of the aliens police evaluate the cooperation activities and decide whether the requirement has been fulfilled. At this point it is in the discretionary power of these to decide, for instance, when an embassy refuses to issue travel documents. Then it has to be assessed whether the individual has a role in that, because he/she has not provided the necessary written or oral information (High level bureaucrat at Viennese agency for social policy, personal communication, 3 October 2016). In the case of confirmed non-cooperation, the BFA sends a notification to the relevant provincial authorities that the person is not cooperating. This notification allows the provinces’ administrations to cut basic welfare support for this individual. The consequences of the notification are not clearly defined by law and by no means consistently applied across the country. In some provinces documented non-cooperation may lead to dismissal, others do not consider non-cooperation as a criterion for barring rejected asylum seekers from basic welfare support; it may just lead to requesting the individual to get in touch with the aliens police (High level bureaucrat at Viennese agency for social policy, personal communication, 3 October 2016; Head of legal counseling for asylum seekers at an NGO in Vienna, personal communication, 14 September 2016). In this instance, regional bureaucrats in the field of welfare decide about the eligibility to social assistance. Therefore, not only different in-country legal provisions exist, but also distinctive interpretations and handlings occur across the provinces.

In conclusion, in Austria the obligation to cooperate is legally defined for both fields, presence and access. However, discretion and implementation make the difference. The federal authority employs a tight interpretation of cooperation with regard to temporary stay, while regional authorities look for leeway to provide basic support and apply the tool in a less strict way.

## Cooperation with return: the case of the Netherlands

As articulated in laws and guidelines, the obligation to cooperate with return is essential to obtain access to a status on the basis of non-removability (*buitenschuldvergunning*) or access to the state-run reception system. At several occasions, CWR was at the centre of conflicts on restrictions in social welfare between the national government and international institutions as well as between the national government and local authorities.

### Cooperation for no-fault status

As an official status for people who cannot be removed, the Netherlands know the no-fault status (*buitenschuldvergunning*). Cooperation is an essential requirement to obtain this status.

Conditions for the no-fault status are stated in law^4^ and require the migrant to take concrete steps to arrange their own departure. For example, by requesting mediation of the Repatriation and Departure Service (DT&V) and International Organization for Migration, by applying to their embassy and by providing the necessary proof of their identity. The migrant is responsible for obtaining the proper documentation to prove his/her identity. Officers of the Immigration Service, in close cooperation with the DT&V, decide if the migrant has sufficiently complied with the given condition and is – despite of his/her efforts – unable to return.

Even though the condition of cooperation is laid down in law, determining whether a migrant has cooperated sufficiently leaves room for discretion by street-level bureaucrats. The Advisory Committee of Migration Affairs (Adviescommissie voor Vreemdelingenzaken [ACVZ], 2013) as well as the Dutch Refugee Council (Vluchtelingenwerk Nederland, 2013) found that it is unclear what exactly is expected of the migrant to comply with the procedure. “In practice, the review of the conditions for granting a *buitenschuldvergunning* (no-fault permit) does not always take place in a uniform and consistent matter and therefore raises concerns” (Adviescommissie voor Vreemdelingenzaken [ACVZ], 2013). A quick scan in 2017 found that the requirements had not been made more explicit in the meantime.^5^

The no-fault status provides access to social welfare, but is granted on a remarkably limited scale. Parliamentary documents show 50–100 requests per year over the last 5 years with a recognition rate of about 20% (Ministry of Security and Justice, 2016b). The vast majority of migrants in limbo therefore cannot access social services through this procedure.

### Cooperation for social benefits

Stratified rights and limitations in access to social services for migrants in limbo are dictated by the Benefits Entitlement Act (*Koppelingswet*), also known as the Linkage Act of 1998. This act amended several laws to link claims for social benefits to a valid residency status. Migrants without a legal residency status are therefore excluded from access to basic services and facilities, such as education, work or financial benefits, as well as food and housing. Exceptions to this act are access to emergency healthcare, access to legal aid and access to education for minors. Following a decision of the High Council (The Netherlands v. Mother and child 1-3, 2012), minors and their families currently have access to shelter and financial benefits in designated Family Locations (Leerkes, 2016; Van der Leun, 2006).

CWR plays an important role in access to state-operated shelter and other basic facilities. Only on the condition that the migrant cooperates with the return procedure will shelter be provided by the national government in a Freedom Restricted Location (VBL). Placement in a VBL is coupled to a freedom-restricting measure under article 56 of the Aliens Act (Legal Advisor Refugee Law, Dutch Refugee Council, personal communication, 3 March 2017). The location is geared towards effectuating a return within 12 weeks, although this can only be partly carried out.^6^ However, if someone continues to cooperate, this period may be extended. “In principle, the alien stays at the VBL for 12 weeks, as long as he is willing to actively work on his return” (Researcher, European Migration Network, personal communication, 3 February 2017).

Cooperation needs to be active and demonstrable. The extent to which the migrant has independently attempted to obtain documents for his or her departure and is willing to provide relevant information is taken into account when assessing cooperation. Case managers of DT&V decide if cooperation has been demonstrated and sufficiently backed with actions. “We [DT&V] always consciously use the word ‘active cooperation with return’ in our texts” (Policy officer, Ministry of Security and Justice and Communications officer, DT&V, personal communication, 16 March 2017). The conditions for access are not stated as an official decree but are drawn up as an administrative, internal guideline.^7^ In practice, as a social rights lawyer points out, it remains vague which actions are required to comply with this condition: “It is all discretionary, it was just a word, it was random. Then when you ask if it is a rule, the answer is no” (Social rights lawyer, Fischer Advocaten, personal communication, 9 February 2017). Reasons for denying or granting access to the VBL therefore vary, which leaves room for discretion of street-level bureaucrats.

Non-compliance with the condition of cooperation leaves migrants in limbo deprived of access to basic facilities. At the same time, compliance may lead to forced or “voluntary” return. CWR, hence, implies contradictory interests for those migrants who may fear to be returned. Some experts working at the intersection of migration and welfare address these ambivalences very openly: “How can you measure willingness to cooperate? It’s a bit of a Freudian construction if you ask me” (Policy advisor migration, Municipality of Utrecht, personal communication, 28 April 2017).

In the Netherlands, the lack of access of migrants in limbo to basic facilities caused tensions between international organizations and the national government on the one side, and between the national government and local governments on the other (Kos et al., 2015). In a decision on immediate measures in 2013, the European Committee of Social Rights (ECSR) urged the Dutch government to “adopt all possible measures with a view to avoiding serious, irreparable injury to the integrity of persons at immediate risk of destitution through the implementation of a co-ordinated approach at national and municipal levels, with a view to ensuring that their basic needs (shelter, clothes and food) are met” (ECSR, 2013). In its decision on the merits in 2014, the ECSR unanimously found that the Netherlands were in violation of the European Social Charter. The ECSR stated that “the provision of emergency assistance cannot be made conditional upon the willingness of the persons concerned to cooperate in the organization of their own expulsion” (ECSR, 2014, p. 18). More recently, the UN Committee on Economic, Social and Cultural Rights repeated the problematic nature of the condition of cooperation for access to basic social services and called upon the government to “refrain from making access to food, water and housing conditional on an individual’s willingness to return to his or her country of origin” (Committee on Economic, Social and Cultural Rights (CESCR), 2017, p. 7).

Especially the considerations regarding conditionality are important in view of this paper. In contrary to the ECSR, the Central Appeals Tribunal and the Council of State decided in 2015 that there is no need to provide emergency assistance, since basic facilities are available in the Freedom Restricted Location. These two courts found that the fact that these facilities are offered on condition of CWR does not mean that basic facilities are insufficiently available. On 5 July 2017, the Council of State confirmed that CWR may be used as a condition for access to shelter, however, it needs to be more clearly motivated (The State Secretary of Security and Justice v. the alien, 2017).

In the meantime, local governments were confronted with non-removed persons in a situation of homelessness and destitution on their streets (Kos et al., 2015; Leerkes, 2016). Backed by the decision on immediate measures by the ECSR, some municipalities opened or expanded shelter facilities. “As a municipality, you are actually not allowed to provide anything (…) We do this on the grounds of public safety and on humanitarian grounds” (Policy officer migration, Municipality of Amsterdam, personal communication, 29 March 2017). Conditions for access to these facilities vary, but do not require the migrant to produce proof of active cooperation with their own expulsion, although in some cases this may lead to added benefits. Municipal facilities are operated without consent of the national government. After a long process of negotiations, the State Secretary of Security and Justice terminated funding for these facilities and expressed its intention to sanction municipalities that continued to offer support (Ministry of Security and Justice, 2016a). Recently, negotiations between specific municipalities and the national government have gradually reopened.

Based on official documents and expert interviews, we conclude that the conditionality of access to basic facilities serves as a tool to demand compliance with migration control policies. The tensions created by the reluctance of certain local authorities to implement CWR strictly illustrate the contradictory interests in the use of this tool (see also Leerkes, Varsanyi, & Engbersen, 2012; Van der Leun, 2006). Below we identify characteristics of the policy tool, present an evaluation on the (modest) effectiveness of the tool with respect to the migration control aim and suggest why this is so.

## Characteristics and effects of cooperation with return

In both countries, the analysis of policy documents as well as the expert interview establish CWR as a key legal and practical condition which has to be fulfilled for access to toleration status and/or basic social services such as housing and financial allowances (primary education and basic health care are exempted from conditionality). However, there are significant differences in the concrete use of the tool in Austria and the Netherlands.

In the first instance, the tool of cooperation appears straightforward, but is in fact very complex in case-to-case implementation. In terms of actors and processes, it does not only involve different layers of governments, but also combines different policy fields. Furthermore, the tool links regulative with distributive, financial goals. In this regard, it follows the stick-and-carrot idea: cooperation with return is the stick, access to status and public services is the carrot. In contrast, non-cooperation is punished and usually leads to a denial of social benefits and/or to detention. Implemented as such, the tool contains ambiguous messages to the affected migrant, it encourages exclusion in the first place, but may lead to (precarious) inclusion in the second.

Considering this simultaneity of different features, it is no surprise that the cooperation tool is vague in definition and weak in realizing the proclaimed goals. Howlett and Ramesh (2003, p. 68) point out that state policies applied in certain sectors may be weak when “authority is dispersed and no one group of officials can take the lead in formulating policy”. In the case of the usage of the policy instrument of cooperation, public officials and agencies involved in deciding on the issue are dispersed across several political levels, agencies and policy chains. As our findings suggest, cooperation is not sufficiently agreed between the two *policy networks* involved. On the one side, welfare aims facilitate well-being in the interests of the individual and society as a whole. Sub-national political and administrative actors follow social interests because of societal problems that exclusionary policies can create. On the other, federal political and administrative actors use CWR for border politics and state sovereignty. National interests to reduce unwanted migration are at the forefront. In this context, our findings show that migration authorities attempt to be strict and restrictive, claiming to act in the interest of the rule of law. In fact, there is a hierarchy between the goals of the two policy fields and their networks, welfare is subordinated to return enforcement goals; the requirement of sufficient cooperation is assessed by migration authorities rather than by welfare authorities. Moreover, there is competence sharing: Federal authorities are in charge of migration control, local and regional officials decide on welfare benefits. Thus, not only two policy fields are at work, but also actors at different political levels form the policy network.

At sub-national *levels*, certain street-level bureaucrats seem to hesitate in implementing the tool strictly. At local level and at the level of NGOs, social services are still provided, although the national level signals restrictions. In Austria, regional officials have internally agreed on a uniform way to deal with the issue to avoid migrants in limbo travelling from one region to another. This is why regional officials and politicians have an interest to keep migrants in limbo within the social system to avoid problems like homelessness, public disorder and destitution (Case worker at an NGO in Vienna, personal communication, 24 October 2016). Sub-national actors largely refrain from connecting welfare too closely with return, but do not talk too much about it in public, instead they negotiate behind the scenes. Decisions by international institutions allowed sub-national actors in the Netherlands to articulate these concerns more openly. These tensions and uncertainties may impact on the direct effects of CWR on the politics of deportation.

When looking at the tool’s *substance* – what kind and degree of cooperation is demanded legally and in practice – we see an interesting difference between the two country contexts. In both countries, a certain degree of discretionary assessment by bureaucrats and case workers is in place about confirming cooperation in exchange for stay and social services. However, these powers are used differently in the two fields and countries. In the Netherlands, access to state-operated facilities is not only de-facto, but also legally coupled with cooperation; national courts have confirmed the justiciability of this condition. In Austria, access to state-run facilities even for those who are not cooperating depends to a certain extent on the discretionary assessment by street-level bureaucrats in regional administrations.

Sainsbury (2006) argues that the type of welfare state has a decisive impact on the scope and quality of immigrants’ social rights. On the basis of our empirical data we argue however, that in the case of social rights of migrants in limbo, it is less the type of welfare state rather than the timing and the degree of *politicization* of irregular migration and the return issue that affects the role of cooperation on access to social rights. Both country studies provide evidence that as soon as the issue has been placed on the political agenda then it becomes regulated and implemented in a more restrictive way. The Netherlands has a long history of politicizing the issue of irregular migration, irregular migrants and welfare (see Rosenberger & Ruedin, 2017). In 1998, the Benefits Entitlement Act was introduced, specifically linking social benefits to a valid residency status and so explicitly excluding all irregular migrants (see Kos et al., 2015). Moreover, in April 2015, the political contestation of access to basic facilities for irregular migrants nearly caused the coalition government to collapse ("Coalition nearly tripped", 2015, inter alia). In Austria, until the general election campaign in 2017, social welfare of migrants pending deportation has not been a political issue at all. In 2004 when the Basic Welfare Support Agreement was introduced, the issue of social support for temporary non-deported asylum seekers was included without any public debate. Although CWR is regulated as one of a few mandatory conditions to be met, until 2016 political parties did not consider this issue relevant. Consequently, we argue that the different extent of politicization directly impacts on the relevance of CWR in the access to social welfare.

In terms of effectiveness, we have to take into account that the policy measure of CWR only concerns migrants who have established a relationship with the state. Those who do not make a claim for social benefits are not influenced by this tool. For the Netherlands, the latest estimate of migrants without legal residency status was made in 2012 by the Scientific Research and Documentation Center/WODC (Van der Heijden, Cruyff, & van Gils, 2015). This study estimates that 35,530 migrants reside there without a legal residency status. This group consists of irregular migrants as well as rejected asylum seekers. Most of the migrants without legal residency status are not “on the radar” of the state and therefore make no claim to social services. For Austria, at first sight, the number of people who do not receive assistance because of non-cooperation is small (for instance: in the province of Upper Austria there are five to six cases; Provincial Minister for integration, personal communication, 7 June 2017). This is no evidence that the tool can tackle the control dilemma in migration. At the same time, however, only a small number of non-deported individuals remain in the basic care system (according to several parliamentary questions and answers, the numbers are around 3000 since 2012, with a slight increase in 2017). Against a deportation gap of roughly of 50–60% (Rosenberger & Küffner, 2016), there is reason to assume that a great number of rejected asylum seekers have absconded and do not apply for social benefits or have gone on to other countries.

These estimated numbers suggest that in both countries the policy tool implies a strong discursive component directed towards the voting population (see Van Oorschot, 2009). It is a signal to the general public that welfare for “unwanted migrants” is provided only on a conditional basis. In fact, it has to be earned individually by demonstrating the willingness to cooperate with the return procedure. The underlying rationale is that only good and needy migrants may be rewarded with basic social services. No doubt the tool aims at clearing away practical barriers against effectuating removals, but the effects are demonstrated less in numbers than in political discourse. Distinct gaps, tensions and ambiguities remain: between immigration control and welfare policy networks, between the stages of legislation and implementation, and between national, sub-national and international actors. These gaps occur, however, in different intensity in the two country contexts.

## Conclusions, limitations and outlook

The paper dealt with migrants in limbo and gave better insights into the relevance and functioning of the policy instrument of CWR. According to national laws, such cooperation with immigration law enforcement authorities must be demonstrated by the individual to earn the right to maintain temporary presence (toleration) and access to basic social services. To pursue the policy goal of clearing practical constraints to non-returnability, policy makers require migrants officially declared unwanted within the territory to collaborate actively with return procedures.

The two study countries present similar contexts of migration and welfare, but they differ in the politicization of the issue of certain immigrant groups. The Netherlands has a legacy of hot political debates on irregular migrants and their access to social benefits since the introduction of the Linkage Act. In Austria, the political contestation has concerned primarily asylum seekers. Only since the year 2016, issues related to the deportation gap have entered the political agenda. Against this background, the findings presented in this paper underline two statements. First, CWR is a more pronounced and explicit tool of migration control in the Netherlands than in Austria. Second, in both countries the tool is (still) more instrumental in addressing a migration sceptical society, than it is functional in tackling the migration control dilemma.

The nature of this policy tool can be characterized as follows: First, it encourages the personalized migration control activities. The state shifts the burden of proof to the individual. Not surprisingly, immigrant communities will generally not have an interest to cooperate, as it limits their possibility to remain in the country. This contradictory situation may lead to people going into hiding and becoming destitute. Additionally, as we have seen above, this may lead to struggles between the national and the subnational level. Second, cooperation is a vaguely defined tool. It implies discretionary leeway on the part of gatekeepers who grant status and welfare provisions, and produces circumstances of contingency, insecurity and indeterminacy on the side of migrants.

Our findings demonstrate that the policy makers’ primary interest in turning to cooperation as a technique serves the aim of signalling state power in times of migration control failures. The instrument is intended to increase effective returns, that is, to remove practical deportation barriers. The question whether and to which extent practical barriers can be removed through this instrument, however, cannot be answered sufficiently by the data available here. We could not find solid evidence that the mix of rewards/penalties would de-incentivize people to remain in cases of a removal order. There is good reason to assume that the control dilemma is not being tackled. However, we conclude that legal and political barriers seem to be more powerful than barriers at the individual level: bilateral agreements, declarations of safe country status/safe area status within countries. To find out the concrete relevance of cooperation for carrying out returns remains therefore a topic for further research.

Cooperation with return is a policy based on the idea to reward, respectively sanction behaviour of individuals. Looking at recent policy developments, there is reason to assume that this policy type will gain greater relevance not only for the return issue but also for reforms within the broader migration/social policy nexus. In Austria, very recently, cooperation has also come up in the policy field of immigrant integration of refugees. The most recent Integration Act (*Integrationsgesetz*) of 2017 foresees that anyone who does not participate, cooperate and complete various trainings offered by the federal state will have their social benefits cut. In the Netherlands, a similar trend can be seen regarding immigrant integration. Recognized refugees have to sign a contract of cooperation with the integration procedure. If a person fails to cooperate by not participating, fines may be imposed. These migration policy developments put the focus on the individual performance rather than on structural frameworks and supportive strategies. Taking up this challenge, this paper provides valuable insights for further analysis of sanction-oriented policy tools which are directed at the performance of individuals while signalling to the wider public a solution to political problems.

Finally, a side effect of the implementation of CWR could be that a considerable group of rejected asylum seekers may go underground, hiding from authorities and not applying for social services at all. This paper could not provide clear answers to the questions, whether and to which extent the tool motivates migrants in limbo to not apply for social benefits because they shy away from helping return authorities by providing identification and travel documents.

Does the tool facilitate non-removable migrants absconding? And what happens in term of social protection with those who do not show up? Thus, more research on the micro-consequences of the cooperation tool for the individual, and on the unintended consequences for the well-being of the whole society is needed.

## Abbreviations

ACVZ: Adviescommissie voor Vreemdelingenzaken
BFA: Bundesamt für Fremdenwesen und Asyl
CESCR: Committee on Economic, Social and Cultural Rigths
COA: Centraal Orgaan opvang Asielzoekers
CWR: Cooperation with return
DT&V: Dienst Terugkeer en Vertrek
ECSR: European Committee of Social Rights
FRA: Fundamental Rights Agency
IND: Immigratie en Naturalisatiedienst
IOM: International Organisation for Migration
NASS: National Asylum Support Service
NGO: Non-governmental organisation
VBL: Vrijheidsbeperkende locatie
VW 2000: Vreemdelingenwet 2000
WODC: Wetenschappelijk Onderzoeks-en Documentatiecentrum

## Footnotes

1 The term *policy instrument* and *policy tool* are used interchangeable in this paper.

2 N.N

3 For example, the Basic Welfare Support Act of Lower Austria (NÖ Grundversorgungsgesetz) states that no entitlement to basic welfare support exists, if „the non-deportability has been brought about by fault of the person seeking help, whereby […] the necessary cooperation to obtain a travelling document has to be assessed in particular (“die Nichtabschiebbarkeit von der Hilfe suchenden Person schuldhaft herbeigeführt wurde, wobei […] insbesondere die erforderliche Mitwirkung zur Erlangung eines Ersatzreisedokumentes […] zu beurteilen ist”) (§3 Abs.2 Nö LGBl. Nr. 80/2015).

4 Vreemdelingencirculaire 2000 (B) par 4.

5 ACVZ, Letter to the Direction Migration Affairs regarding Quick scan *buitenschuldbeleid*, 9 October 2017.

6 According to research by investigative journalists for the programme Argos in 2016, 45 out of the 350 residents in the VBL stayed there for longer than one year.

7
https://www.dienstterugkeerenvertrek.nl/VertrekuitNederland/LeidraadTerugkeerenVertrek/index.aspx
